# Eculizumab for the treatment of glycine receptor antibody associated stiff-person syndrome

**DOI:** 10.1007/s00415-023-11777-0

**Published:** 2023-05-18

**Authors:** Jennifer A. McCombe, Bryan T. Klassen, Eoin P. Flanagan, James W. Teener, Anastasia Zekeridou, Sean J. Pittock, Andrew McKeon

**Affiliations:** 1 Division of Neurology, Department of Medicine, University of Alberta, Edmonton, Alberta Canada; 2 Department of Neurology, Mayo Clinic, Rochester, Minnesota USA; 3 Department of Laboratory Medicine and Pathology, Mayo Clinic, Rochester, Minnesota USA; 4 Department of Neurology, University of Michigan, Ann Arbor, Michigan USA

Dear Sirs,

Glycine receptor alpha 1 subunit antibody (GlyRα1-IgG) is detected in some patients with stiff-person syndrome (SPS) spectrum, most commonly progressive encephalomyelitis with rigidity and myoclonus (PERM) [1–3]. GlyRα1-IgG (primarily IgG1) in vitro activates the complement cascade on the cell surface of live human embryonic kidney (HEK) 293 cells expressing GlyR-α1 [1]. Therefore, there is possibly a role for complement-directed therapies in the treatment of GlyRα1-IgG-associated SPS. There is also concern for serious opportunistic infection to potentially arise during complement inhibition due to encapsulated bacteria, particularly meningococcus [4]. We present two patients treated with eculizumab, a monoclonal antibody therapy which binds C5 and prevents its cleavage to C5a and C5b. Written informed patient consent was obtained.

An 18-year-old woman with a history of epilepsy presented with episodic tingling, followed by jerk-like movements of the head, shoulder, and arm, sometimes spreading down her spine to her leg. The movements occurred unilaterally, alternating sides, at a maximum of 30 times daily. They occurred spontaneously or were triggered by startle. There was no altered awareness. Back pain and tightness accompanied the episodes. Escalation of anti-seizure medication was ineffective. The movements were recorded without EEG electrographic correlate.

Examination at Mayo Clinic revealed reduced arm swing, increased limb tone, and hyperreflexia throughout. Electrophysiologic testing revealed abnormal startle response to acoustic stimulus, with spread of muscle activity to all muscles tested, and no habituation with repeat stimuli. CSF evaluation was normal. Antibody testing in serum detected GlyRα1-IgG by cell-binding assay. GlyRα1-IgG was not detected in CSF.

Following diagnosis, multiple immunotherapies were trialed, including IVIg, plasmapheresis, rituximab, mycophenolate, and azathioprine, with incomplete improvement in symptoms. Ultimately, disease control was achieved with plasmapheresis every 10 days, rituximab every 3 months and azathioprine 50 mg twice daily. She noted worsening of symptoms with any widening of the plasma exchange treatment interval. The patient also required symptomatic treatment with clonazepam, 0.5 mg TID. The patient desired pregnancy and so alternative treatments were considered, and eculizumab was initiated, after elimination of other immune suppressants (though plasma exchange was retained). Over the ensuing months, she tapered off plasma exchanges and maintained complete symptomatic control on eculizumab alone. Repeat electrophysiological testing 1 year after continued treatments with eculizumab revealed normalization of acoustic startle response.

A 20-year-old man was diagnosed with stiff-trunk variant SPS at age 11. At that time, he presented with subacute onset back pain and spasms, culminating in ICU admission. One year after onset, GlyRα1-IgG was detected in serum and he was treated with steroids and IVIG, with minimal response, followed by plasmapheresis and rituximab, with some benefit and reduction in symptomatic therapies. Over many years he received immunotherapy trials with mycophenolate, azathioprine, belimumab and IVIg, although achieved the best symptom control with rituximab. Despite this, the patient required significant ongoing symptomatic therapies with undesirable side effects, and typically had worsening symptoms with B-cell repopulation. Given incomplete disease control, alternative treatments were considered and eculizumab was initiated.

The patient received rituximab 13 months before initiation of eculizumab, and still had undetectable B-cells. The patient received tetravalent meningococcal vaccine against serogroups A, C, W, and Y and meningococcal vaccine against serogroup B. Given ongoing B-cell suppression, he received meningococcal prophylaxis with penicillin. Response to vaccine was not tested.

Two months after initiating eculizumab, the patient, without neurological improvement, travelled to Central America. One week after returning, he developed headache, vomiting, fever, and rash. CSF microbiology revealed N. meningitidis with intermediate penicillin sensitivity. Following ceftriaxone treatment, he had a rapid resolution of meningitis. Treatment with eculizumab was not re-initiated.

GlyRα1-IgG associated autoimmunity results in varying clinical phenotypes including progressive encephalomyelitis with rigidity and myoclonus (PERM) and SPS. Multiple reports have confirmed clinical phenotype and typical response to immunotherapy [1, 2, 5]. Neuronally-expressed glycine receptors in brainstem and spinal cord are accessible to potentially pathogenic IgGs for downregulation (internalization) [1, 3]. This supports an approach to therapy consisting of first line treatment with IVIg or plasmapheresis with or without steroids, followed by an anti-B-cell therapy. Given the demonstration of C3b deposition on GlyR–transfected cells [1], a role for anti-complement therapy could be considered in patients not responsive to initial therapies.

In the first patient, GlyRα1-IgG was not detected in CSF. As previously reported, many patients have antibody detected in serum but not in CSF, particularly in those patients SPS-spectrum patients without PERM [1]. It has been noted that patients with clinical presentations in keeping with PERM may have GlyRα1-IgG detected in CSF more often than patients presenting with symptoms of SPS [1].

Eculizumab is a terminal complement inhibitor approved for treatment of both acetylcholine receptor antibody positive myasthenia gravis (AChR-IgG positive MG) and aquaporin-4 antibody positive neuromyelitis spectrum disorder (AQP4-IgG positive NMOSD) [6]. Common side effects of eculizumab include infusion reactions (such as hyper/hypotension, rash, and fever) and opportunistic infection. Of particular concern, is the risk from encapsulated bacterial infection and in particular Neisseria species, notably Neisseria meningitidis. Eculizumab is likely safe during pregnancy as drug levels found in umbilical cord samples demonstrate levels insufficient for affecting complement levels in the newborn [7], providing our patient with options for maintaining disease control during conception, pregnancy, and, if desired, breast feeding, as it is also undetectable in breastmilk.

The second patient suffered a serious complication despite appropriate vaccination and antibiotic prophylaxis, prior to determining if eculizumab was effective. Meningococcal meningitis is a recognized complication of anti-complement therapies including eculizumab; thus CDC recommends vaccination against this prior to initiation of eculizumab [7–9]. Despite this, 16 cases of meningococcal disease in eculizumab recipients were identified in the US between 2008 and 2016, representing an increased risk of meningococcal disease by 2000-fold [8]. In Japan, 17 patients died of suspected meningococcal disease over 3559 person-years of eculizumab therapy, representing 6100 times greater than baseline risk of meningococcal disease [10]. It appears as though vaccination alone may not provide adequate protection, which may relate to prior exposure to immune suppression, in particular B-cell therapies, blunting response to vaccination, or continued risk may be due to eculizumab mediated inhibition of killing of meningococcus despite vaccination [11, 12]. As such, some clinicians, recommend analysis of serologic response to vaccination with antibiotic prophylaxis (penicillin or ciprofloxacin) until this is documented [4, 13]. Others still, and indeed some regulatory bodies, advocate for the use of ongoing antibiotic prophylaxis while prescribed eculizumab [4]. However, as this case demonstrates, even for patients who are vaccinated and receiving meningococcal prophylaxis, antimicrobial resistant forms of N. meningitidis, prevalent in some regions, still present a risk [14].

The associated risk of disseminated meningococcal diseases underscores consideration of this treatment for GlyRα1-IgG associated SPS only after failure of other, more commonly used, immunotherapies. We would generally recommend trial of steroids ± IVIg/plasmapheresis as first line treatment with consideration of second line treatments including rituximab, azathioprine and mycophenolate. Only when these therapies have been trialed, with treatment failure defined as intolerance, continued symptom progression, or ongoing need for multiple symptomatic therapies, would we consider eculizumab.
